# The Risk Factors That Predict Chronic Hypertension After Delivery in Women With a History of Hypertensive Disorders of Pregnancy

**DOI:** 10.1097/MD.0000000000001747

**Published:** 2015-10-23

**Authors:** Ji-won Hwang, Sung-Ji Park, Soo-young Oh, Sung-A. Chang, Sang-Chol Lee, Seung Woo Park, Duk-Kyung Kim

**Affiliations:** From the Division of Cardiology, Department of Internal Medicine, Cardiovascular Imaging Center, Cardiac and Vascular center, Samsung Medical Center, Sungkyunkwan University School of Medicine, Seoul, Republic of Korea (J-WH, S-JP, S-AC, S-CL, SWP, D-KK); and Department of Obstetrics and Gynecology, Samsung Medical Center, Sungkyunkwan University School of Medicine, Seoul, Republic of Korea (S-YO).

## Abstract

Hypertensive disorders of pregnancy (HDP) is one of the most important lethal complications in pregnant mothers. It is also associated with the subsequent development of chronic hypertension. The objective of this study was to identify the clinical risk factors of postpartum chronic hypertension in women diagnosed with HDP.

Six hundred patients as HDP, who diagnosed and followed-up at least 6 month after delivery, were included in the study. We divided the included subjects in 2 groups based on the development of postpartum chronic hypertension: presenting with the chronic hypertension, “case group” (n = 41) and without chronic hypertension, “control group” (n = 559).

Clinical and demographic factors were evaluated. By multiple regression analysis, early onset hypertension with end-organ dysfunction, smoking, higher prepregnancy body mass index (BMI), and comorbidities, systemic lupus erythematosus (SLE) or antiphospholipid syndrome (APLS), were associated with progression to chronic hypertension in the postpartum period. The value of area under the curves (AUC) for the 5 models, that generated to combine the significant factors, increased from 0.645 to 0.831, which indicated improved prediction of progression to the chronic hypertension. Additional multivariate analysis revealed significant specific risk factors.

This retrospective single hospital-based study demonstrated that the clinical risk factors, that is early onset hypertension with end-organ dysfunction, smoking, and higher prepregnancy BMI, were significant independent predictors of chronic hypertension in women after delivery. Identification of risk factors allowed us to narrow the subject field for monitoring and managing high blood pressure in the postpartum period.

## INTRODUCTION

Hypertensive disorders of pregnancy (HDP) is an important parturition-associated disorder with lethal effects on mothers and children.^1^ High blood pressure (BP) in pregnant women is associated with pulmonary edema, stroke, acute kidney injury, disseminated intravascular coagulopathy, and death in the antepartum period.^2^ There has been extensive research on the risk factors, incidence, pathogenesis, prevention, and management of HDP mainly in the antepartum period.^3^ It is also important that these complications are controlled in the postpartum period. Women with pregnancy-induced hypertensive disorder have an increased risk of essential hypertension, stroke, other vascular diseases, end-stage renal disease, and diabetes mellitus, later in life.^4–9^ Pregnancy is now considered as an important risk for future hypertension, cardiovascular disease, and metabolic disease.^10,11^

Postpartum hypertension is a common cause for concern, similar to hypertension in the antepartum period. Hypertension that persists to the postpartum period can threaten well-being and longevity in life. Controlling the high BP during antepartum and postpartum periods is related to reduced maternal morbidity and mortality.^3^ Likewise, HDP is strongly correlated to subsequent chronic hypertension or essential hypertension.^4^ Hypertension itself is an important risk factors in cardiovascular disease. Hence, it is important to study the association of HDP and subsequent chronic hypertension and identify the predictors of high BP, for overall improvement of cardiovascular health.

Currently, it is difficult to ascertain to what extent this concern is justified and there are few available guidelines for clinical management. There is very little information on the clinical risk factors of chronic hypertension at the postpartum period. Until now it has been difficult to predict the clinical outcome of chronic hypertension.

The objective of this study was to evaluate the potential predictors of progression to chronic hypertension in the women diagnosed with HDP.

## METHODS

### Study Population

A retrospective cohort study was performed on Korean women with the diagnosis of HDP who were admitted and delivered, between January 2005 and September 2012 at Samsung Medical Center. The discharge summary and EMR of each woman was reviewed by a single investigator (JWH) based on ICD-9, to determine cases diagnosed with HDP.

The inclusion criteria for the HDP were: systolic BP (SBP) ≥140 mm Hg or a diastolic BP (DBP) ≥90 mm Hg, measured on at least 2 occasions during gestation, with the regular follow-up at the pregnancy period; at least 6 month after delivery during the postpartum period, clinical followed-up at the same center. It was the status of consistent high blood pressure during this following period in these pregnant patients. We also included the patient with the sign of end-organ dysfunction (ie, kidney, liver, and blood cell count) although normotensive state. Exclusion criteria were: referrals to other hospitals without management or delivery in this center for the individuals’ reasons, or no postpartum follow-up. In addition, the patients were also excluded, who had comorbidities.

The 97 cases, who diagnosed as other comorbidities, were excluded, such as renal disease (including the glomerulonephritis, autosomal dominant polycystic kidney disease, the status of postkidney transplantation, and certain chronic kidney diseases); heart disease (including angina pectoris with medication, valvular heart disease, and atrial septal defect); diabetes mellitus (DM); vascular disease (including Takayasu's arteritis, moyamoya disease, and renal artery stenosis).

The outcome was chronic hypertension, which was defined as persistent high-BP state >6 months after delivery when confirmed to the record of hospital visit. Although most women with new onset hypertension in pregnancy become normotensive after delivery, some cases remain hypertensive for >6 months postpartum.^12^ We decided this point to the criteria of dividing the case and control groups. The patients with the case group were identified as chronic hypertension with persistent high BP >6 months after delivery and maintained on antihypertensive medications in the postpartum period for > 6 months.^12^

This study was approved by the Human Research Ethics committee of the Samsung Medical Center and was conducted according to the Declaration of Helsinki.

### Classification of Hypertensive Disorders of Pregnancy

According to the currently accepted definition of the International Society for the Study of Hypertension in Pregnancy, women with HDP have gestational hypertension, pre-eclampsia, and high BP in the first trimester together with eclampsia, HELLP syndrome.^2,13^

Gestational hypertension is defined as new hypertension with an SBP ≥140 mm Hg or a DBP ≥90 mm Hg on 2 separate occasions, without proteinuria, arising de novo after the 20th week of pregnancy. Pre-eclampsia meets the following conditions: proteinuria (>0.3 g during 24 h or at least 2 + of protein on dipstick testing) and de novo hypertension such as gestational hypertension after the 20th gestation week. Early onset hypertension with end-organ dysfunction meets the following conditions: de novo hypertension before the 20th week and proteinuria, or evidence of headache, blurred vision, abdominal pain, low platelets, or elevated liver enzymes. We have used this terminology and classification with reference to these 3 disorders throughout the text.^13^

### Established Clinical Risk Factors and Variables

We selected a series of clinical and demographic parameters, with known association with hypertension and which could be easily acquired from pregnant women.^4,12^ Maternal age at diagnosis with HDP, prepregnancy body mass index (BMI), history of smoking before pregnancy, the mode of delivery, the number of the pregnancy (gravida) at HDP, and some laboratory findings that reflected the organ function such as platelet, total bilirubin, liver enzymes, creatinine, and proteinuria in the point of detecting the HDP.^12,14–18^ We included these variables as potential clinical risk factors among the established risk factors.

These parameters were confirmed by the medical records obtained on history-taking and laboratory test results of patients. In 1 patient, the first detection date of HDP was at the baseline of the deciding factors.

Comorbidities were maternal medical conditions such as renal disease (including the glomerulonephritis, autosomal dominant polycystic kidney disease, the status of post-kidney transplantation, and certain chronic kidney diseases); heart disease (including angina pectoris with medication, valvular heart disease, and atrial septal defect); diabetes mellitus (DM); abnormal status of thyroid function (including hypothyroidism or hyperthyroidism); systemic lupus erythematosus (SLE); antiphospholipid syndrome (APLS); vascular disease (including Takayasu's arteritis, moyamoya disease, and renal artery stenosis); and hematologic disease (including idiopathic thrombocytopenic purpura, paroxysmal nocturnal hemoglobinuria, myelodysplastic syndrome, aplastic anemia, and lymphoma). Among these conditions, the renal disease, heart disease, DM, and vascular disease were initially excluded. The mode of delivery was recorded as either spontaneous delivery or Cesarean section: gravida as either primi or multi. Other factors such as smoking and twins were recorded as the presence or the absence.

### Statistical Analysis

The case and control groups of the patients were compared according to the factors included in the analysis. Maternal age at diagnosis of HDP, prepregnancy BMI, and some laboratory data (platelet, bilirubin, liver enzymes, and creatinine) were treated as continuous variables. Gestational hypertension, pre-eclampsia, early onset hypertension with end-organ dysfunction, comorbidities, smoking, the mode of delivery, twin, the number of the pregnancy (meaning gravida) at HDP, and proteinuria were ordered as categorical variables. Continuous variables are presented as mean ± standard deviation (SD) and categorical variables as absolute numbers and frequency percentages. Continuous variables were compared using the Student *t* tests and categorical variables were compared using the χ2 tests or Fisher's exact.

Multiple logistic regression analysis with the significant variables (*P* < 0.05) on univariate analysis was conducted to choose the independent clinical predictors of postpartum hypertension. The adjusted relative risk of each predictor was evaluated by multivariate analysis. The odds ratio (OR) and 95% confidence interval (CI) were calculated.

Receiver-operating characteristic (ROC) analysis was conducted by combining these significant predictors in multivariate analysis as an adjunct method. The area under the curves (AUCs) was generated to identify cutoff values (defined as those with greatest sensitivity plus specificity sum) through the respective models, adding these factors 1 at a time.

All statistical analyses were performed with SPSS (version 20.0, SPSS Inc, Chicago, IL) and Medcalc (version 9.6) and a *P* value <0.05 was considered statistically significant.

## RESULTS

### Baseline Characteristics

Finally, 600 enrolled patients were included in the 2 groups. The included subjects were further divided into case and control groups.

The case group (n = 41, 6.83%) included patients diagnosed as chronic hypertension at the postpartum period. The control group (n = 559, 93.17%) comprised the rest of the study subjects. A total of 600 patients in this study were characterized with unadjusted associations of established risk factors with subsequent chronic hypertension in the postpartum period as shown in Table 1. Hypertensive disorders of pregnancy was classified with gestational hypertension, pre-eclampsia, and early onset hypertension with end-organ dysfunction. There were statistically significant differences in pre-eclampsia and early onset hypertension with end-organ dysfunction. Of the 600 patients, 41 (6.83%) patients were diagnosed with chronic hypertension after delivery. The women in the case group were older (33.32 ± 4.55 vs 32.02 ± 3.88, *P* = 0.041) and had higher prepregnancy BMI (23.98 ± 5.10 vs 21.87 ± 3.43, *P* = 0.013), compared to the control group. Any laboratory findings were not significantly different in 2 groups. There was the significant difference in the number of the pregnancy (primi vs multi gravida) at HDP. Gestational age of fetus (a fetal factor considered) was statistically different between the case vs control groups (232.58 ± 40.91days vs 250.44 ± 25.61 days, respectively, *P* = 0.011).

**TABLE 1:**
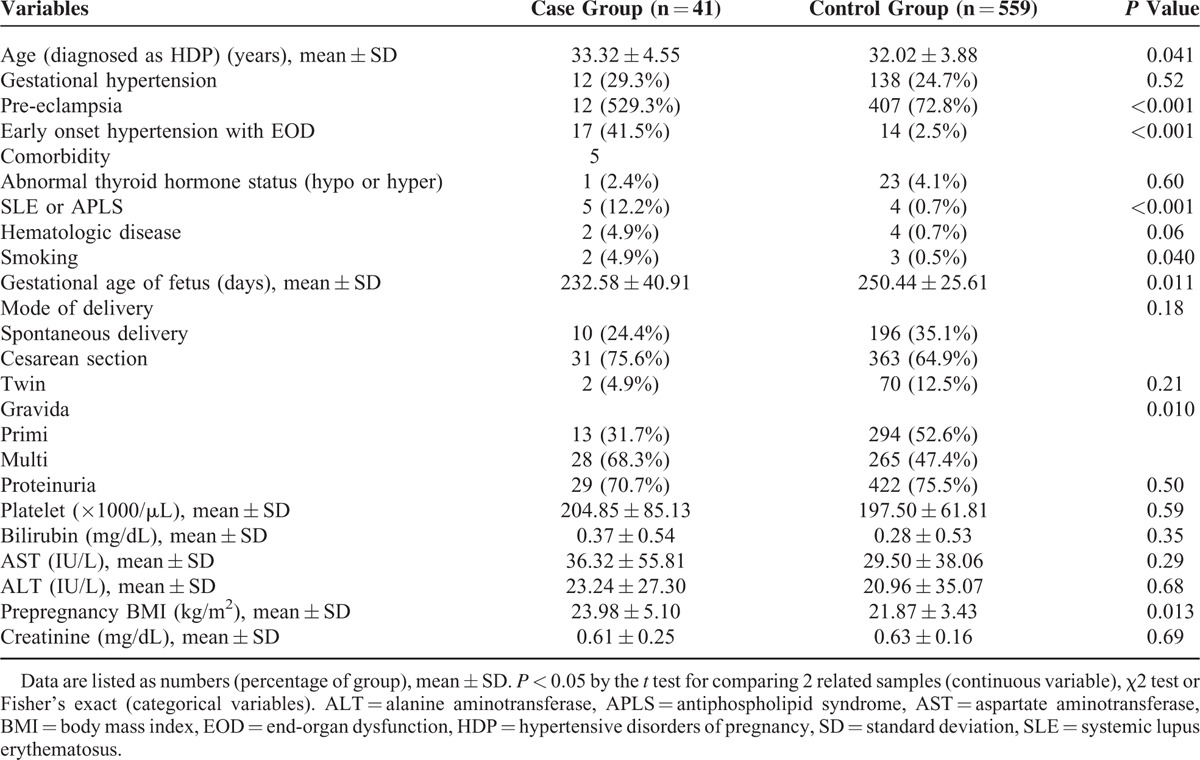
Basic Characteristics of the Study Group

### Predictors of Progression of the Chronic Hypertension

**FIGURE 1:**
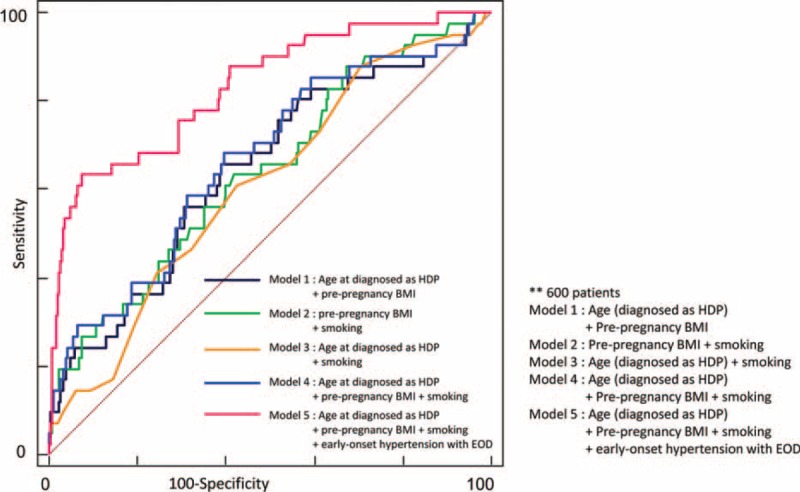
Comparison of ROC analysis in the models respectively. ROC = receiver-operating characteristic.

In multivariate regression analysis, early onset hypertension with end-organ dysfunction, history of smoking, and prepregnancy BMI were predictors of progression to chronic hypertension (Table 2). The incremental pattern of AUCs for the 7 models showed that the prediction of subsequent chronic hypertension improved, to add the significant risk factors to the multivariate regression analysis (Table 3, Figure 1). We used a series of multiple logistic regression models to estimate the adjusted odds ratio of chronic hypertension in the postpartum period. In model 1, we adjusted for maternal age at HDP and prepregnancy BMI. In model 2, we included the variable of adjusted prepregnancy BMI and smoking. In model 3, we included maternal age at HDP and smoking. In model 4, we combined with maternal age at HDP, prepregnancy BMI, and smoking. And then, in model 5, we added early onset hypertension with end-organ dysfunction to model 4 for adjusting. The value of AUCs was used to check the incremental effect of predictors of progression to chronic hypertension in the respective models. These results are presented as follows: model 1 with 2 adjusted risk factors (AUC of model 1 = 0.645). Area under the curves of model 5 with the factor of early onset hypertension with end-organ dysfunction added was higher than model 1 (AUC of model 5 = 0.831). Area under the curves was similar with model 1 to 4 (from 0.609 to 0.666).

**TABLE 2:**
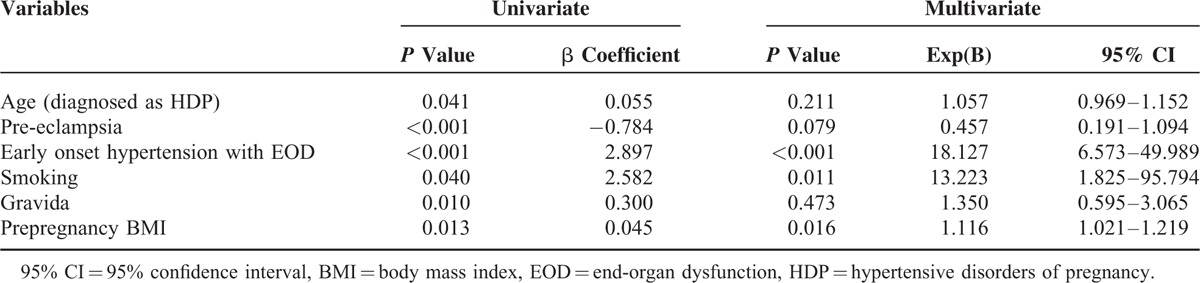
Univariate and Multivariate Analyses of Determinants of Chronic Hypertension in HDP Patient

**TABLE 3:**
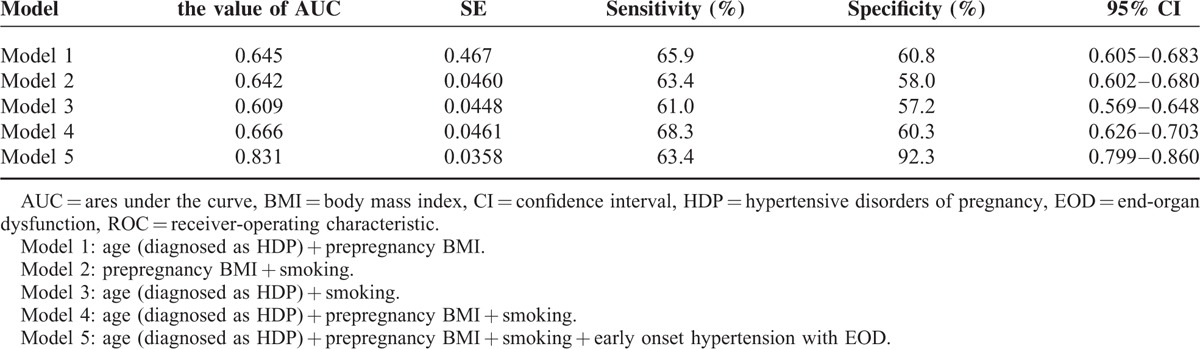
Comparison of Receiver-Operating Characteristic (ROC) Analysis in the Models Respectively

## DISCUSSION

The main finding of our study was that in patients with a diagnosis of HDP, so the clinical risk factors of early onset hypertension with end-organ dysfunction, history of smoking, prepregnancy BMI were independent predictors of progression to chronic hypertension.

### Clinical Importance of Hypertensive Disorders of Pregnancy

Hypertensive disorders of pregnancy, a new-onset hypertension during pregnancy, is a common problem, and its consequence on future maternal health must be carefully evaluated. Hypertension is an important predisposing factor to cardiovascular disease, and HDP is definitely associated with the future chronic hypertension as previously reported.^4,11^ Recent data demonstrated that elevated blood pressure during pregnancy, regardless of type and risk factors, signals high risk of later cardiovascular disease, chronic kidney disease, and diabetes mellitus. The striking finding was that any history of hypertension during pregnancy was associated with a higher risk of subsequent hypertension, despite the absence of prepregnancy risk factors, such as obesity and smoking.^19^ Thus, it is imperative for the clinicians to recognize risk factors and accordingly determine the patients to be monitored. Until now there was limited data on the development of subsequent chronic hypertension. This study has introduced the possibility of regulating fatal effects of HDP for the mother's better long-term health, particularly hypertension, and cardiovascular disease. Previously, there were a few studies including only small numbers of patients belonging to the Korean-Asian race. Another study of a Dutch population^20^ had an ∼25%. Total prevalence of chronic hypertension after HDP in Dutch population was higher than our data, that is, 11% chronic hypertension after delivery. The difference may be related to race, Dutch and Asian, or it is also likely that our data was underestimated in comparison as 8% patients of new-onset hypertension after delivery were included in the Dutch study.

It is preferable to subdivide HDP as case managements differed with each classification.^2^ In addition, the likelihood of subsequent to chronic hypertension was dependent on the classification of HDP. The women, who had the above risk factors such as smoking and obesity, would need to receive the appropriate treatment from a collaborative team of other specialists together with obstetricians. Also all clinical conditions that reflect the status of pregnant women were evaluated in order to predict future hypertension. These included the maternal age at HDP, the history of smoking, the mode of delivery, twins, the number of the pregnancy (meaning gravida) at HDP, higher prepregnancy BMI, and some laboratory findings. Summarily, the details of these conditions associated with pregnancy will be translated to patient general care. In terms of associated fetal factors, the shorter gestational age of fetus was the result of severity and timing of onset of HDP.

We showed that clinical risk factors, noted at the time of pregnancy, were predictors of subsequent chronic hypertension. The predictive capacity had incremental increases as certain postpartum factors were added on. The strong risk factors were early onset hypertension with end-organ dysfunction, prepregnancy BMI, and smoking.

Our findings suggests that clinicians should determine the occurrence of risk factors in all pregnant women and accordingly guide lifestyle modification such as weight reduction, and regularly BP monitoring, with continued care through the postpartum period, and future by their other medical providers. The medical providers have a role providing timely patient education, continuous monitoring of signs and symptoms, and prompt and appropriate case management. Our results also indicate that the patients would need ongoing hypertension management provided by cardiologists or nephrologists in the associated divisions as well as possible long-term antihypertensive medications. The one of the strengths in this study was evaluated in the case group while early excluded the comorbid illness as confounding factors because of controlling the influence of outcome.

The risk factors we identified were easy to assess and handle in the clinical field. Furthermore, the prevalence of metabolic modifiable risk factors such as obesity, and high BMI was high at postpartum screening. The results suggest that lifestyle intervention would be beneficial to the women with chronic hypertension after delivery. Additionally, our data suggests that these factors and HDP are a good population screening tool during the postpartum period. Our study clearly showed the relationship between chronic hypertension and the defined classification of elevations in BP and proteinuria, over the course of pregnancy. Thus, the emphasize lies in the importance of time of detection and severity of hypertension and proteinuria during pregnancy. Our data also showed an association between gestational age of fetus and maternal pre-eclampsia.

### Study Limitation

Our study has several limitations. First, it was a retrospective single hospital-based cohort and small case group study. Second, we could not consider all gravida cases in 1 patient, for instance, 1 patient could be pregnant several times and the condition of pregnancy, occurrence of HDP and subsequent chronic hypertension or not could potentially be different on all occasions. Hence, in order to try to overcome this limitation, the first diagnosis of HDP was configured as the standard in this study because there were some cases that 1 patient was pregnant more than once. Early onset hypertension with end-organ dysfunction is a valuable predictor for chronic hypertension. The classification of HDP is subdivided according to the degree of accompanying organ failure such as thrombocytopenia, proteinuria, azotemia, and elevated hepatic enzymes. Laboratory tests were hence reviewed at that time of first diagnosis of HDP. There was some discrepancy of HDP and laboratory data in the univariate analysis. For example, although proteinuria is the important point of classifying gestational hypertension and pre-eclampsia, proteinuria data was not a significant value in univariate analysis.

Moreover, this study had some selection bias due to inclusion of a tertiary hospital for monitoring blood pressure and receiving prescriptions. We could not follow up and monitor all patients with diagnosed subsequent chronic hypertension during long term. The presence or the absence of chronic hypertension could not be ascertained in these cases because of loss of follow-up. The loss of follow-up occurred in a considerable number of patients. Finally, the number of cases with eclampsia or HELLP syndrome was too small, precluding the possibility of determining the association with these 2 criteria. In future studies, it might be possible to estimate the correlation between these 2 classifications and chronicity of hypertension in particular.

## CONCLUSIONS

Some women with diagnosed HDP became normotensive whereas others remained continuously hypertensive and progressed to chronic hypertension in the postpartum period. We showed that some clinical and demographic risk factors such as early onset hypertension with end-organ dysfunction, higher prepregnancy BMI, and smoking were reliable predictors of progression to chronic hypertension in the postpartum period. In each patient, the hypertensive state was presumed to be maintained and clinicians accordingly determined strict control of the high BP. Patients with associated clinical factors were also closely monitored.

Further large population-based prospective studies, with long-term follow-up, need to be conducted to determine the important risk for ischemic cardiovascular and cerebrovascular diseases among women who had new-onset hypertensive disorders of pregnancy.

## References

[1] Hutcheon JA Lisonkova S Joseph KS Epidemiology of pre-eclampsia and the other hypertensive disorders of pregnancy. *Best Pract Res Clin Obstet Gynaecol* 2011; 25:391–403. 21333604 10.1016/j.bpobgyn.2011.01.006

[2] Jim B Sharma S Kebede T Hypertension in pregnancy: a comprehensive update. *Cardiol Rev* 2010; 18:178–189. 20539101 10.1097/CRD.0b013e3181c60ca6

[3] Sibai BM Diagnosis and management of gestational hypertension and preeclampsia. *Obstet Gynecol* 2003; 102:181–192. 12850627 10.1016/s0029-7844(03)00475-7

[4] Callaway LK Mamun A McIntyre HD Does a history of hypertensive disorders of pregnancy help predict future essential hypertension? Findings from a prospective pregnancy cohort study. *J Hum Hypertens* 2013; 27:309–314. 23223085 10.1038/jhh.2012.45

[5] Wilson BJ Watson MS Prescott GJ Hypertensive diseases of pregnancy and risk of hypertension and stroke in later life: results from cohort study. *BMJ* 2003; 326:845. 12702615 10.1136/bmj.326.7394.845 PMC153466

[6] Bellamy L Casas JP Hingorani AD Pre-eclampsia and risk of cardiovascular disease and cancer in later life: systematic review and meta-analysis. *BMJ* 2007; 335:974. 17975258 10.1136/bmj.39335.385301.BE PMC2072042

[7] Melchiorre K Sutherland GR Liberati M Preeclampsia is associated with persistent postpartum cardiovascular impairment. *Hypertension* 2011; 58:709–715. 21844489 10.1161/HYPERTENSIONAHA.111.176537

[8] Fraser A Nelson SM Macdonald-Wallis C Associations of pregnancy complications with calculated cardiovascular disease risk and cardiovascular risk factors in middle age: the Avon Longitudinal Study of Parents and Children. *Circulation* 2012; 125:1367–1380. 22344039 10.1161/CIRCULATIONAHA.111.044784 PMC3323835

[9] Lykke JA Langhoff-Roos J Sibai BM Hypertensive pregnancy disorders and subsequent cardiovascular morbidity and type 2 diabetes mellitus in the mother. *Hypertension* 2009; 53:944–951. 19433776 10.1161/HYPERTENSIONAHA.109.130765

[10] Kaaja RJ Greer IA Manifestations of chronic disease during pregnancy. *JAMA* 2005; 294:2751–2757. 16333011 10.1001/jama.294.21.2751

[11] Sattar N Greer IA Pregnancy complications and maternal cardiovascular risk: opportunities for intervention and screening? *BMJ* 2002; 325:157–160. 12130616 10.1136/bmj.325.7356.157 PMC1123678

[12] Podymow T August P Postpartum course of gestational hypertension and preeclampsia. *Hypertens Pregnancy* 2010; 29:294–300. 20670153 10.3109/10641950902777747

[13] Davey DA MacGillivray I The classification and definition of the hypertensive disorders of pregnancy. *Am J Obstet Gynecol* 1988; 158:892–898. 3364501 10.1016/0002-9378(88)90090-7

[14] Samuels-Kalow ME Funai EF Buhimschi C Prepregnancy body mass index, hypertensive disorders of pregnancy, and long-term maternal mortality. *Am J Obstet Gynecol* 2007; 197:490 e491–496. 17714679 10.1016/j.ajog.2007.04.043 PMC2100395

[15] Stepan H Nordmeyer AK Faber R Proteinuria in hypertensive pregnancy diseases is associated with a longer persistence of hypertension postpartum. *J Hum Hypertens* 2006; 20:125–128. 16239899 10.1038/sj.jhh.1001952

[16] Peterson E Craigo S House M Risk factors for postpartum antihypertensive medication requirement in severe preeclampsia. *Hypertens Pregnancy* 2010; 29:350–356. 20670157 10.3109/10641950902968700

[17] Morikawa M Cho K Yamada T Risk factors for postpartum hypertension in women with twin pregnancies. *J Perinat Med* 2012; 40:115–120. 22017329 10.1515/JPM.2011.113

[18] Sibai BM Etiology and management of postpartum hypertension-preeclampsia. *Am J Obstet Gynecol* 2012; 206:470–475. 21963308 10.1016/j.ajog.2011.09.002

[19] Mannisto T Mendola P Vaarasmaki M Elevated blood pressure in pregnancy and subsequent chronic disease risk. *Circulation* 2013; 127:681–690. 23401113 10.1161/CIRCULATIONAHA.112.128751 PMC4151554

[20] Spaan JJ Sep SJ van Balen VL Metabolic syndrome as a risk factor for hypertension after preeclampsia. *Obstet Gynecol* 2012; 120:311–317. 22825090 10.1097/AOG.0b013e31825f21ff

